# Monitoring the Structural Health of Glass Fibre-Reinforced Hybrid Laminates Using Novel Piezoceramic Film

**DOI:** 10.3390/s20185428

**Published:** 2020-09-22

**Authors:** René Schmidt, Alexander Graf, Ricardo Decker, Michael Heinrich, Verena Kräusel, Lothar Kroll, Wolfram Hardt

**Affiliations:** 1 Professorship of Computer Engineering, Chemnitz University of Technology, Straße der Nationen 62, 09111 Chemnitz, Germany; wolfram.hardt@informatik.tu-chemnitz.de; 2 Professorship for Forming and Joining, Chemnitz University of Technology, Reichenhainer Straße 70, 09126 Chemnitz, Germany; alexander.graf@mb.tu-chemnitz.de (A.G.); verena.kraeusel@mb.tu-chemnitz.de (V.K.); 3 Department of Lightweight Structures and Polymer Technology, Chemnitz University of Technology, Reichenhainer Straße 31/33, 09126 Chemnitz, Germany; ricardo.decker@mb.tu-chemnitz.de (R.D.); michael.heinrich@mb.tu-chemnitz.de (M.H.); slk@mb.tu-chemnitz.de (L.K.)

**Keywords:** hybrid assembled composite, piezoceramic compound, fibre metal laminates, fatigue, signal processing

## Abstract

This work investigates a new generation structural health monitoring (SHM) system for fibre metal laminates (FML) based on an embedded thermoplastic film with compounded piezoceramics, termed piezo-active fibre metal laminate (PFML). The PFML is manufactured using near-series processes and its potential as a passive SHM system is being investigated. A commercial Polyvinylidene fluoride (PVDF) sensor film is used for comparative evaluation of the sensor signals. Furthermore, thermoset and thermoplastic-based FML are equipped with the sensor films and evaluated. For this purpose, static and dynamic three-point bending tests are carried out and the data are recorded. The data obtained from the sensors and the testing machine are compared with the type and time of damage by means of intelligent signal processing. By using a smart sensor system, further investigations are planned which the differentiation between various failure modes, e.g., delamination or fibre breakage.

## 1. Introduction

Non-destructive testing (NDT) is used to detect failures inside of polymer based lightweight structures, but this is usually only carried out offline and involves complex and cost-intensive methods, e.g., ultrasonic testing [1], eddy current testing [2], infrared thermography [3], laser shearography [4], and X-ray radiography [5]. Therefore, NDT methods are mainly used to detect manufacturing defects or damage. However, the future trend is to use these tools for the evaluation of the material condition and the prediction of the remaining life time of the structure [6]. In order to meet these goals, several SHM systems already allow online measurements by structure-integrated or -applied sensors, which are divided into the following sensor types.

- Resistance strain gauges [7,8]
- Fibre optic sensors, e.g., fibre Bragg grating (FBG) strain sensors [9,10,11]
- Active or passive piezoelectric sensors [12,13,14]
- Sensors using the electrical properties: resistance, impedance, dielectric, etc. [15,16]

The monitoring of the structural condition of lightweight structures can be carried out either by local sensor placement at positions with a high inclination to initial damage or by remote placement of the sensor in order to carry out an area measurement. Depending on the physical measurement principle and the type of structural monitoring, NDT methods can be divided into passive detection and active detection of structural integrity. The passive method only monitors structural/material properties or the occurrence of a damage event, whereas the second method actively interrogates the structure to derive a comprehensive structural behaviour.

New approaches of passive sensing are based on online-capable SHM systems that combine advanced sensor technologies with intelligent algorithms and thus allow a diagnosis of the structural integrity of the monitored component [17,18]. In addition to improving component reliability and safety, maintenance intervals can be extended and maintenance costs reduced.

The use of such SHM systems is particularly relevant for classic highly stressed structures in mobile applications (transportation, rail vehicles, aerospace, etc.), which are increasingly being replaced by hybrid material systems based on metal/plastic pairings [19]. The aim is to combine the high strength and stiffness properties of metal with the high design freedom of plastics [20]. With the targeted use of the “right” material at the “right” place, damage tolerance and damping can be significantly increased compared to the individual components [21]. In addition, such material systems offer particular advantages in terms of mass reduction, as they are highly flexible in adapting their characteristic properties to the loads acting on the component. The main disadvantages of this non-inherent material combination are the strongly varying material properties and the discontinuous material interfaces, which often induce failure/critical stress conditions in the area of the boundary layer under load.

With the increasing use of such hybrid material systems, however, production- and application-related requirements for the use of SHM systems are also changing. While less installation space is available for additional functions due to increasingly complex assemblies, the demand for increased function density is growing. To solve this contradiction, it is necessary to integrate a minimum number of discrete sensors into the structure, which monitor the structural integrity over a wide area [17,22].

A preliminary study showed that the PFML has potential for use as an SHM system, after which an approach for the feasibility of spectral energy density (SED) was investigated. It was observed that the SED of an undamaged PFML increases slightly independent of force and dispersion, whereupon the energy signals were analysed in more detail by means of descriptive statistics. It could be shown that a reproducible and characteristic trend of the standard deviation is obtained. The preliminary study was a proof of concept with a small number of samples and was therefore not published. Building on this, the present study analyses the signals with regard to a validation of the PFML as an SHM system. The PFML consists of a thermoplastic piezoelectric material which, in combination with the FML, creates a passive sensor system and therefore forms an integral part of the component. The basis for the mostly required large-scale production is thus a PFML, which is manufactured in a continuous production process [17,18,22].

## 2. Materials and Methods

### 2.1. Materials and Specimen Preparation

**Figure 1:**
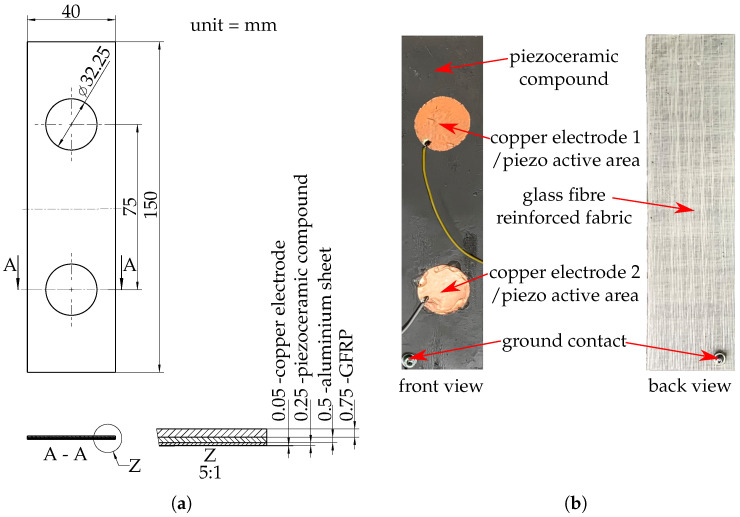
Prepared specimen (**a**) drawing; (**b**) front and back view.

The test specimens in this work are asymmetric hybrid laminates as shown in Figure 1. They consist of three different layers:

- an aluminium sheet (EN AW-6082 T4)
- a piezo-active sensor layer
- a reinforcement layer of glass fibre-reinforced plastics (GFRP)

Two different types of sensor layers are investigated. The first type of sensor layer is based on a piezo-active compound, which is made from polypropylene (Moplen HP501H (LyondellBasell Industries N.V., Rotterdam, The Netherlands)) filled with 70 wt.% piezoceramic lead zirconate titanate (PZT) powder (NCE55 (CTS Corporation, Lisle, IL, USA)) and 0.5 wt.% carbon nanotubes (PLASTICYL PP2001 (Nanocyl S.A., Sambreville, Belgium)) [23,24]. This compound is processed into a thin film with a thickness of 250 μm by means of film extrusion and calendering technology. In a subsequent rolling process, the film is joined with the aluminium sheet [25]. Two copper electrodes are applied to the pizoceramic film and the specimens are polarised by a high-voltage electrical field to activate the piezoelectric effect [26,27]. In detail, the specimens were polarised with an electric field strength of 4.5 $\frac{\mathrm{kV}}{\mathrm{mm}}$ in a dielectric oil bath at 125 °C for a duration of 5 min. For reference purposes, specimens with a second sensor type are prepared. For this purpose, two film patches of polyvinylidene fluoride (PVDF; DT4-028K (TE Connectivity Ltd., Schaffhausen, Switzerland)) are applied to the aluminium sheet instead of the piezoceramic film and the copper electrodes. In all specimens, the two active areas have a diameter of 32.25 mm and an area of 4.25 cm${}^{2}$. They are placed at a distance of 75 mm.

After functionalisation with the sensor layer, the aluminium sheet is combined with the reinforcement layer made of GFRP. It has a fibre volume content of ~45% and consists of bidirectionally aligned 0${}^{{}^{\circ}}$/90${}^{{}^{\circ}}$ glass fibre fabrics. In this work, two different matrix materials are compared. For this purpose, thermoset epoxy resin (EP) and thermoplastic polypropylene (PP) are used.

After joining the functionalised aluminium sheet with the reinforcement layer and curing the specimens (epoxy: 24 h at RT), they are cut to the outline dimensions of 150 mm × 40 mm. The average thickness is approximately 1.5 mm. Figure 1 shows the prepared test specimen as well as the composition and thickness of the layer structure. Altogether, four different specimen configurations were prepared:

- aluminium-based fibre metal laminate with PZT/PP film sensor layer and glass fibre epoxy reinforcement (PFML-PZT-EP)
- aluminium-based fibre metal laminate with PZT/PP film sensor layer and glass fibre polypropylene reinforcement (PFML-PZT-PP)
- aluminium-based fibre metal laminate with PVDF film sensor and glass fibre epoxy reinforcement (PFML-PVDF-EP)
- aluminium sheet with PZT/PP film sensor layer, but without fibre-reinforcement as reference specimen for static mechanical testing (AL-PZT)

The studies in this work started with the investigation of the PFML-PZT-EP specimen. This was the laminate type of the preliminary tests. Based on this, the active material of the sensor layer and the matrix material of the reinforcement layer were varied.

### 2.2. Experimental Procedure

**Figure 2:**
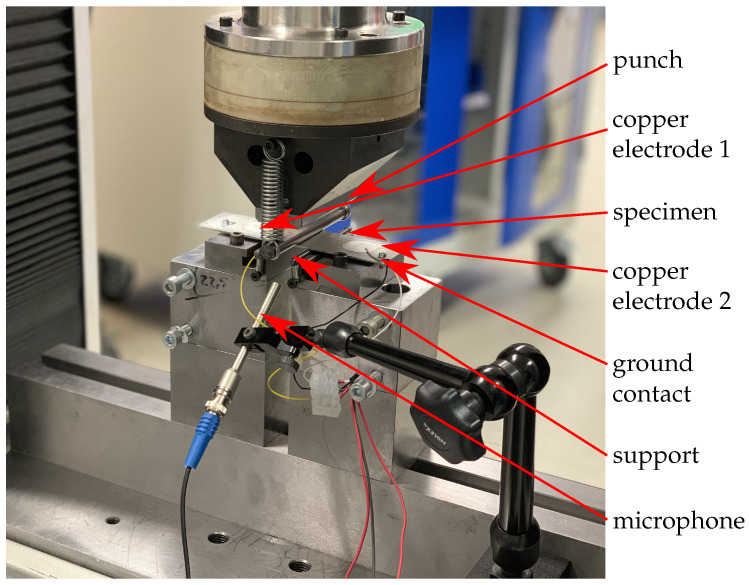
Set-up of the static and dynamic three-point bending test.

The tests for static and dynamic testing of the laminates were carried out using a three-point bending test. The experimental set-up consists of a punch with a radius R = 5 mm and two supports with a radius r = 2 mm. The distance L between the supports is determined by:

(1) $$\begin{array}{c} L=16\cdot t \end{array}$$

and results for the average laminate thickness t = 1.5 mm in is L = 24 mm. The parameters are based on the ASTM D7774-17 [28] standard. All tests were carried out in a laboratory at a constant temperature of 22.5 °C. An overview of the test set-up is shown in the Figure 2. It can be seen that the specimen was positioned with the sensors facing upwards. The reason for this is that if the sensors are facing downwards, they can have electrical contact with the supports and therefore significantly disturb the signal. To record the signals, the right and left sensor are wired and the ground contact is attached to the specimen. For clear identification of the damage, the acoustic signals are also recorded during the measurement for selected specimens with a microphone of type M360 (Microtech Gefell GmbH, Gefell, Germany, frequency range: 20 Hz to 20 kHz). The microphone is positioned 72 mm from the centre of the specimen. Furthermore, the forces and displacement are measured via the universal testing machine. The destroyed specimens are then visually evaluated and assigned to the failure types, fracture or delamination.

A total of three studies were conducted. The first, a case study, was the preliminary test to determine the applicability of the piezoceramic film. The second study was performed without audio measurement and the third was a repetition of the second study, but with audio measurement to be able to reference the signals clearly. The second and third study are the focus of this publication; they are further divided into a static and a dynamic test. For each of the three laminate types, three specimens were used for the static test and five specimens for the dynamic test. From the PFML-PVDF-EP test series, three of the static and one of the dynamic tests were conducted with audio measurement. In the dynamic PFML-PZT-EP and PFML-PZT-PP dynamic test series, the audio signals of two specimens in each series were recorded. In total, 24 specimens were investigated in studies 2 and 3.

**Figure 3:**
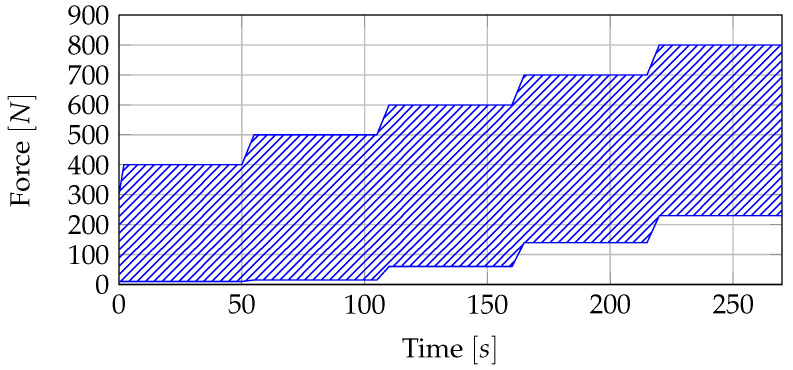
Predefined sequence for the dynamic test in the testing machine.

In the static test series, the punch moves in a path-controlled mode until the specimen fails. The test is aborted by the machine if the force change is less than 70% of the last force value. In the dynamic test series, a cyclical force curve was specified, which is shown in Figure 3. To simplify matters, only the upper and lower envelope curve and the test range are shown. All cycles were run at a frequency of approximately 2 Hz. In total, the test sequence consists of five stages (400 N, 500 N, 600 N, 700 N, 800 N). The beginning is the stage with 400 N. When the load is released, the force never returns to zero to prevent unintentional movement of the specimen. The transition to the next higher force is also completed cyclically. It is known from the case study that some specimens can fail even with a linear increase in force. To determine the exact point of failure, the transition was also described by linearly increasing cycles. Each of the five stages goes through 100 cycles. The tests are stopped manually if the damage has progressed so far that the specimens can no longer absorb any force.

### 2.3. Data Recording

During mechanical testing, the mechanical load, the specimen deformation, the signals of the sensor layer and the acoustic emissions of the specimen are recorded. The punch force and the deflection of the specimen during the three-point bending test are recorded directly by the universal testing machine with a sampling rate of 50 Hz. The two integrated sensors of the test specimen, the additional microphone and the force output of the universal testing machine are connected to a data acquisition system (CS-7008, imc Test & Measurement GmbH, Berlin, Germany). The two sensor channels and the microphone channel are recorded synchronously with a sampling rate of 50 kHz, while the force signal is recorded with a sampling rate of 100 Hz.

As the data was recorded independently on the universal testing machine and the data acquisition devices with individual start times and sampling rates, data synchronisation is required. To achieve a comparable sampling rate, all recorded data from the universal testing machine and the data logger were interpolated to the sensor sampling rate of 50 kHz. Afterwards, the time offset of the signals was determined based on the force signal since this was recorded on both machines. To determine the time offset, the cross-correlation of the interpolated force signals and the offset of the global maximum were determined. Subsequently, the summed absolute difference of the force signals of both determined delays was calculated and represents the decision criteria for the correct time delay estimation. Consequently, all signals are displayed with a sampling rate of 50 kHz and synchronised by the force signal.

### 2.4. Signal Characteristics

**Figure 4:**
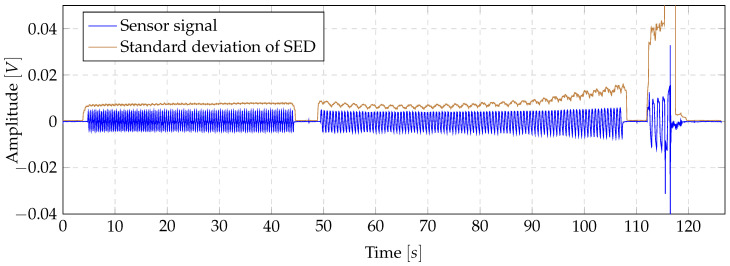
Characteristic of the standard deviation of the SED found in the preliminary study in comparison to the normalized sensor signal.

In a first preliminary study, the possible use of the novel thermoplastic-based fibre-metal laminate in combination with spectral energy density (SED) was analysed for applications in the SHM sector. It was observed that the SED concentrated in a certain range independent of force and dispersion while the object is structurally healthy. For this reason, the energy signals were analysed using descriptive statistics as in state-of-the-art methods using the SED for structural health monitoring applications with piezoelectric sensors [29,30,31]. In some cases a characteristic progression of the standard deviation was found, as shown in Figure 4. As the case study included four specimens, no meaningful statement could be deduced as to whether this method is suitable for representing the health status of the specimen or whether this only occurs for a specific type of damage. However, these results prompted the further studies, which led to the motivation to analyse the SED feasibility for SHM in detail.

To analyse the feasibility of the SED, the sensor signal *S* was decomposed into measurement windows of size $2^{n}$, resulting in a set of measurement windows $S=\{S_{1},S_{2},\ldots ,S_{k}\}$ where *k* represents the maximum number of measurement windows. Each measurement window $S_{j}$ is represented as:

(2) $$S_{j}=\left\{s_{j,1},s_{j,2},\ldots ,s_{j,2^{n}}\right\}$$

**Figure 5:**
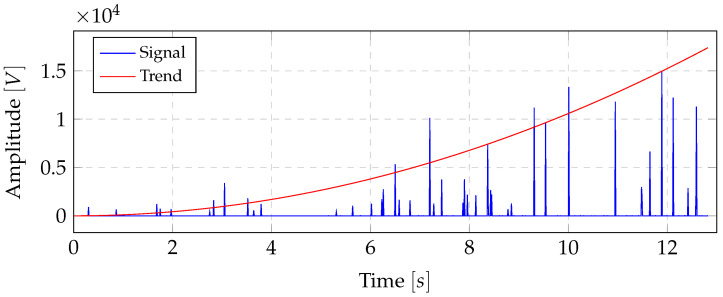
Example of peaks forming a characteristic trend.

The SED $P_{j}$ of measurement window *j* is determined by:

(3) $$P_{j}=\frac{1}{2^{n}}\sum_{f=0}^{2^{n}}X_{f^{2}}^{j}$$

where $X_{f}^{j}$ represents frequency band *f* of the Fourier transform of measurement window $S_{j}$. To reduce the leakage effect, a Hamming window was applied before the Fourier transformation. Subsequently, the standard deviation of the resulting vector *P* was calculated using a sliding window of size 50. Furthermore, using the normalized SED leads to a lack of realisation of global intensity changes [32], which suggests a non-normalised use of the SED. The analysis of the standard deviation of the SED was examined across all specimens. For this purpose, the characteristic progression of the resulting standard deviation function was analysed if at least one sensor per specimen showed a characteristic trend, as found in the preliminary study (cf. Figure 4) or a such a trend could be estimated by peak connections, as shown exemplarily in Figure 5. The formation of such a characteristic trend is the basis for predictability of the remaining lifetime of the laminate and thus represents an important property for the advanced SHM systems. In addition, it was analysed whether the complete failure of the laminate led to a significant peak on at least one sensor per specimen. The formation of significant peaks allows efficient detection by comparison with a threshold value. Furthermore, a threshold algorithm can be efficiently implemented on embedded devices, providing the possibility of mobile real time damage detections.

The accuracy of the signal processing results depends on the window size $2^{n}$, since this directly affects the resolution of the frequency bands as a result of Fourier transform. Furthermore, in many domains it is state of the art to specify the power density spectrum in decibels. The associated logarithmic scaling of the SED can have both positive and negative effects, since the weighting of smaller magnitudes of the frequency bands is greater in relation to larger magnitudes. This can provide positive effects if a constant intensive signal, like the noise of the universal testing machine, is applied to the signal. On the other hand, important intensity differences of the magnitudes can be cancelled out due to the compression effect. Due to this reason, the analysis is performed as a function of the window size $2^{n}$, with n∈{8,9,…,15} and the used scaling of the power spectrum, which varies between decibel (dB) and linear scale, resulting in a total of 16 configurations per specimen.

However, most state-of-the-art methods use the wavelet packet-based energy index [33,34,35] for various applications like monitoring timber connection [36], railway axles [37] or wood utility poles [38] and for fibre-reinforced laminated composites [39,40]. In addition, Song et al. describes a damage index representing the proportion of transmission energy losses caused by damages based on continuous wavelet transform (CWT), which has been successfully applied to bridge bent-caps as an indicator of the health state [32]. Consequently, the damage index as well as the standard deviation of the SED measure the energetic deviation from the normal healthy state.

A breaking of the glass fibres creates a vibration in the laminate, which in turn results in an energy change. This energetic change leads to a change in the statistical distribution of the measured values, which is quantified by the standard deviation allowing the difference from the normal state of the laminate to be observed. Furthermore, the resulting reduced stiffness of the specimens leads to a global increase in the voltage change in the piezoactive areas due to the increased bending of the laminate and thus to an increased energy level as long as no deformation occurs. The damage index methodology is comparable because it measures the energetic deviation from the normal state of the specimen caused by the same damage based vibrations. For this reason, the damage index is included in the analysis as a further indicator for the usability of the thermoplastic-based fibre-metal laminate for the SHM sector. The calculation of the damage index for measurement window $S_{j}$ is based on the root-mean-square deviation calculation represented by:

(4) $$I_{j}=\sqrt{\frac{\sum_{i=1}^{2^{n}}{\left(E_{j,i}-E_{h,i}\right)}^{2}}{\sum_{i=1}^{2^{n}}\left(E_{h,i}^{2}\right)}}$$

where $E_{j,i}$ describes the energy at measurement window *j* and in the frequency band *i* calculated on the basis of CWT. $E_{h,i}$ represents the energy in the healthy state of the specimen. Contrary to the implementation of Song et al. [32], who use one measurement window in the healthy state, this study calculates $E_{h,i}$ as mean value of the first 10 % of the overall measurement windows for each frequency band *i*. This adjustment is necessary due to the different specimen types used. The bridge bent-caps have a much higher potential energy leading to less natural vibration production, whereas the thermoplastic-based fibre-metal laminates used in this study represent lightweight specimens that tend to produce natural vibration, averaged in this way. The energy $E_{j,i}$ of measurement window *j* of frequency band *i* is calculated by the CWT decomposition [35,41] of $S_{j}$, where $Y_{j}=\left\{y_{j,1},y_{j,2},\ldots ,y_{j,m}\right\}$ leading to the definition:

(5) $$E_{j,i}=Y_{i}^{2_{2}}=y_{j,1}^{2}+y_{j,2}^{2}+\ldots +y_{j,m}^{2}$$

In this study, the Daubechies wavelet base db9 is used for CWT, leading to non-overlapping measurement windows due to the orthogonality of the Daubechies wavelet base. Since the same wavelet base was used by Song et al. [32], the comparability of the results is ensured. Since the accuracy of the CWT is influenced by the measurement window size similarly to the Fourier transformation, the analysis of the complete failure detection as well as the trend formation is carried out as a function of the window size analogous to the standard deviation of the SED analysis.

From the previous description, the following hypotheses are derived for the further investigation of the feasibility of the novel thermoplastic-based fibre-metal laminate in the field of SHM:

**Hypothesis** **1** **(H1).** The standard deviation of the SED indicates the time of complete failure.

**Hypothesis** **2** **(H2).** The standard deviation of the SED results in a characteristic trend formation.

**Hypothesis** **3** **(H3).** The standard deviation of the SED indicates the damage type.

**Hypothesis** **4** **(H4).** The damage index indicates the time of complete failure.

**Hypothesis** **5** **(H5).** The damage index serves as an indicator for the health status of the specimen.

## 3. Results

For clarification and comparison of the tests, the results are divided into the sub-chapters static and dynamic tests. For comparison purposes, the maximum force $F_{max}$ and the maximum achievable bending $s_{max}$ distance are listed. In addition, a reference consisting only of an aluminium specimen with a layer of piezoceramic film is added. This means that the unreinforced specimen is the reference. The strengthening value, *S*,

(6) $$\begin{array}{c} S=\frac{F_{max,Al-GFRP}}{F_{max,Al}} \end{array}$$

describes the effect of fibre-reinforcement ($F_{max,Al-GFRP}$) in comparison to aluminium ($F_{max,Al}$) and the ductility, *D*,

(7) $$\begin{array}{c} D=\frac{s_{max,Al-GFRP}}{s_{max,Al}} \end{array}$$

describes the effect of the fibre-reinforcement ($s_{max,Al-GFRP}$) on the maximum bending distance compared to unreinforced aluminium ($s_{max,Al}$). Both variables are used to represent the effect of hybrid laminates. The dynamic tests are compared using the maximum force before failure and the maximum number of cycles. The following analysis results were obtained jointly for the specimens from studies 2 and 3.

### 3.1. Static Three-Point Bending

#### 3.1.1. Mechanical Characteristics

An overview of the specific mechanical properties of thermoplastic and thermoset laminates is given in Table 1. It lists the maximum force achieved, the maximum bending distance and reinforcement as well as ductility. The piezoceramic film and the PVDF sensors were used as sensors for this test series. When comparing the reinforcement, the thermoplastic laminate withstands 2.5 times the force of the unreinforced sheet metal. In return, the ductility decreases. Compared to the thermoplastic laminates, the thermoset laminates can absorb twice as much force (Table 1). On the other hand, the ductility of thermoset laminates is only a quarter of that of the reference and also three times lower than that of thermoplastic laminates. In this test series, both sensor types (PVDF and piezoceramic film) were used. The results show that the sensor type has no influence on the maximum force or the maximum bending distance.

**Figure 6:**
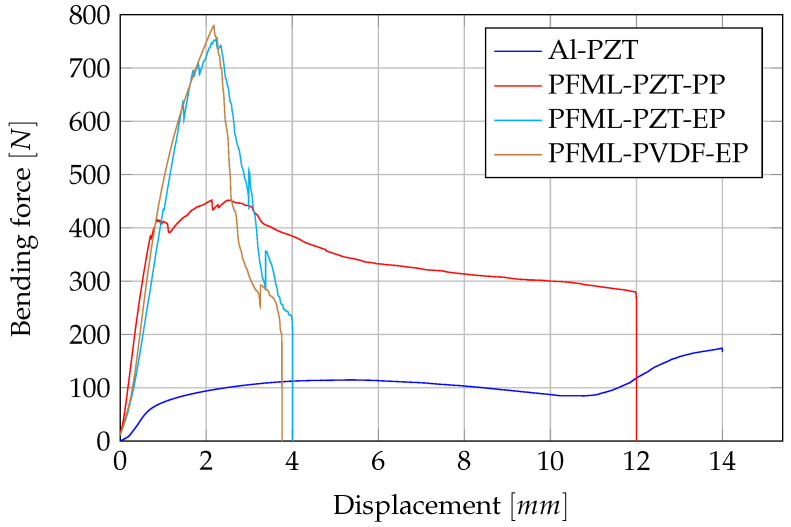
Comparison of the force–displacement curves from the static three-point bending test.

The comparison of all specimen types can be seen in the force–displacement diagram (Figure 6). As described above, the thermoset specimens show a steep increase up to a relatively high force and then suddenly fail due to fibre tearing. In contrast, the thermoplastic laminates fail rather ductile and can maintain the force at an almost constant level over a large bending distance. This is a typical result for thermoplastic fibre-reinforced plastics and shows their good damage resistance properties. The unreinforced aluminium specimen exhibits a typical force–displacement curve when bent. There is a flat increase to maximum, followed by a slight decrease in force and finally another increase in force due to increased friction with the supports.

**Figure 7:**
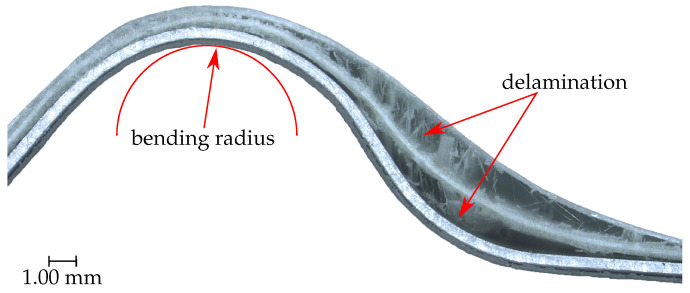
Failure of the thermoplastic laminate after the static three-point bending test.

The failure of the two laminate types under static load is very different. This was recorded using a light microscope. The thermoplastic laminate fails mainly due to delamination in the bending legs (Figure 7). It is noticeable that the failure occurs not only at the interface between GFRP and metal, but also within the GFRP. The failure location is typical for delamination, as this is where the greatest shear stresses act.

**Figure 8:**
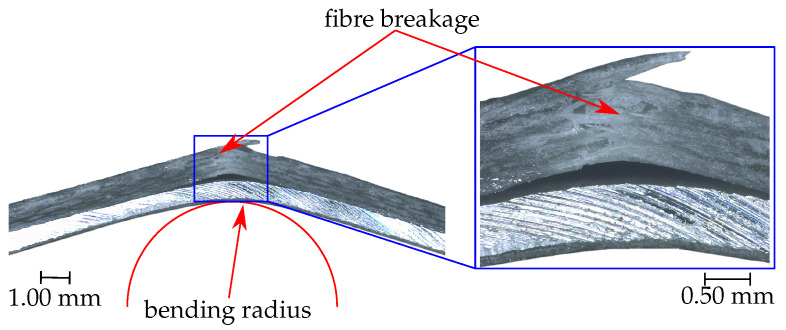
Failure of the thermoset laminate after the static three-point bending test.

The failure of the thermoset laminates is mainly fibre cracking, which is shown in Figure 8. The failure location is exactly in the bending zone where the greatest tensile stresses occur. The crack passes through the entire GFRP layer and causes a partial delamination between metal and GFRP. A positive feature of both laminates is that the piezoceramic film does not tear or delaminate at any time.

#### 3.1.2. Signal Characteristics

##### Complete Failure Analysis Results

The analysis results of the standard deviation of the SED regarding a significant peak formation at the moment of complete failure for the static tests are shown in Table 2. The results show that for each laminate at least one configuration is achieved which forms a significant peak at the moment of complete failure. Furthermore, the best results are achieved with a measurement window size of 2048 and 4096, where just one specimen of the PFML-PZT-EP does not show a significant peak at the time of complete failure using the decibel scale. Additionally, the decibel scale always provides similar or better results than the linear scale, except for the laminate PFML-PZT-PP with measurement window size of 256 and 512, where no specimen formed a significant peak at the time of complete failure. Furthermore, no significant peaks are formed at window sizes 16,384 and 32,768 due to by a too short test duration. These two large window sizes in combination with the sliding window size of 50 for the calculation of the standard deviation result in long observation times of 16.384 s and 32.768 s, representing a longer measurement time than the whole static test duration. The scaling analysis show the feasibility of the decibel scale, since for each laminate one window size leads to a significant peak formation at the moment of complete failure for all specimens. Conversely, the linear scaling does not lead to a significant peak formation in one case of the hybrid laminate PFML-PZT-EP independent of the measurement window size. However, the laminates PFML-PVDF-EP and PFML-PZT-PP do form a significant peak for all specimens by using linear scaling for almost all measurement window sizes.

With regard to the damage index, it can be seen that this forms a significant peak for all specimens and window sizes from 256 to 2048 at the time of complete failure. Furthermore, the damage index results of laminate PFML-PVDF-EP show significant peak formation for all samples in all window sizes, except 32,768. On the contrary, one specimen of the laminates PFML-PZT-EP and PFML-PZT-EP with window sizes larger than 4096 does not form a significant peak at the time of complete failure.

##### Characteristic Trend Formation Analysis Results

The trend results of the formation analysis are shown in Table 3 for the static tests. In therms of the standard deviation of the SED, none of the specimens formed a trend characteristic for window sizes 256 to 1024, except one specimen of laminate PFML-PZT-PP. Furthermore, the applicable window size for trend formation depends on the laminate used, since no measurement window size provides high trend formation results for all laminates examined. All specimens of laminate PFML-PVDF-EP only form a trend with window size 8192 and decibel scale use. Considering all configurations, only one specimen of laminate PFML-PZT-EP formed a characteristic trend at window size 4096 independent of the scaling used, and window size 8192 with decibel scale use. Laminate PFML-PZT-PP achieves the best results with decibel scale use and a window size of 4096 to 16,384.

On the other hand, the damage index forms a trend for all specimens for laminate PFML-PVDF-EP and PFML-PZT-PP, while just one specimen of laminate PFML-PZT-EP forms a characteristic trend in the best case. For both analysed methodologies, damage index and standard deviation of the SED, no best suited window size for characteristic trend formation can be determined, since PFML-PVDF-EP achieves the best results with a measurement window size greater than 1024, and PFML-PZT-PP at window size 512.

##### Complete Failure Type Analysis Results

Taking into account the complete failure type of the laminate specimens used in the static tests, it is seen that all specimens of PFML-PZT-PP are delaminations, while all specimens of PFML-PZT-EP result in fibre breakages. Laminate PFML-PVDF-EP delaminated once, while two specimens show fibre breakages. In terms of complete failure detection, no significant difference can be observed, since many configurations in all investigated samples lead to significant peak formation at the moment of complete failure. On the other hand, trend analysis shows that the delaminated specimen of laminate PFML-PVDF-EP forms a trend only at window size 8192 and standard deviation of the SED use, independent of the scaling. However, laminate PFML-PZT-PP forms no trend by linear scaling and standard deviation of SED use, but two out of three specimens form a characteristic trend at window size 4096 to 16,384 using the decibel scale. In case of laminate PFML-PZT-EP, only one specimen forms a characteristic trend at window size 4096 with linear scaling and at window sizes 4096 and 8192 with decibel scale use.

The damage index forms a characteristic trend for the delaminated specimens of laminate PFML-PVDF-EP at all window sizes, except 4096. Furthermore, laminate PFML-PVDF-EP forms a characteristic trend for all specimens with fibre breakages at window sizes 1024 to 16,384. Including the results of laminates PFML-PZT-PP, all delaminated specimens form a characteristic trend at window size 512 and damage index use, which cannot be observed for the fibre breakages when considering the results of PFML-PZT-EP results.

### 3.2. Dynamic Three-Point Bending

#### 3.2.1. Mechanical Characteristics

The dynamic tests were compared in terms of the maximum number of cycles that can be achieved and the associated force applied. Both, thermoplastic and thermoset laminates were used as specimens. In the case of the thermoset laminates, a distinction was made between the sensor type, piezoceramic film and PVDF (Table 4). The thermoplastic laminates fail in the dynamic test already after the first stage at a force of 400 N and after 234 cycles. The failure is mainly delamination between aluminium and fibre-reinforced plastic. This is due to poor adhesion between the two components. By using a thermoset matrix, higher forces and a greater number of cycles can be achieved in the dynamic test. This is due to the better adhesion of the matrix to the aluminium. The failure of the thermoset laminates is mainly fibre breakage, but some exceptions also fail by delamination.

**Figure 9:**
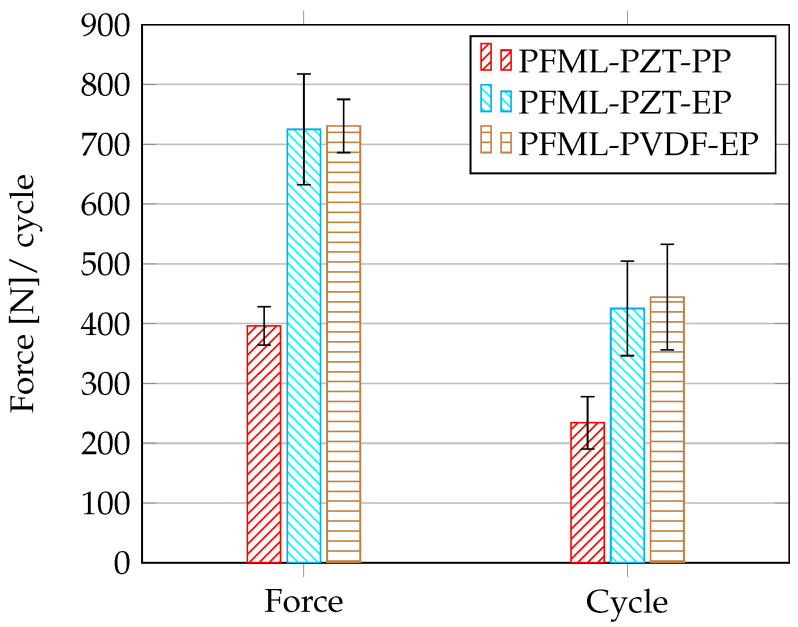
Comparison of the maximum values: force and cycles from the dynamic three-point bending test.

To compare all specimen types, the results are shown in a bar chart for maximum force and the maximum cycles (Figure 9). As mentioned, the dynamic behaviour of the thermoplastic laminates is worse than that of the thermoset laminates.

In this experiment, no influence of the sensor type on the dynamic behaviour was found.

#### 3.2.2. Signal Characteristic

##### Complete Failure Analysis Results

The results of the dynamic tests for complete failure detection are shown in Table 5. The standard deviation of the SED evaluation of the complete failure detection of laminates PFML-PVDF-EP and PFML-PZT-EP shows a significant peak formation in almost all configurations for all laminate, independent of the scaling used. The only two exceptions are window size 256 for PFML-PZT-EP and window size 32,768 for PFML-PVDF-EP. In the first case, linear scaling generates a significant peak for all specimens, but decibel scaling fails for one specimen. In the second case, no significant peak can be detected in one specimen regardless of the scaling used.

In contrast, laminate PFML-PZT-PP forms, in the best case, a significant peak at the moment of complete failure in four out of five specimens with linear scaling, independent of the measurment window size used. In contrast to the results obtained with laminates PFML-PVDF-EP and PFML-PZT-EP, laminate PFML-PZT-PP forms in forms significant peaks in fewer samples at the moment of complete failure when decibel scaling is used compared to linear scaling.

The analysis of the damage index shows that all configurations of laminate PFML-PZT-EP show a significant peak at the moment of complete failure. In contrast, the amount of failed peak formations increases for laminates PFML-PVDF-EP and PFML-PZT-PP at window sizes larger than 2048. In the range of window sizes from 256 to 2048, the damage index forms a significant peak in all cases for laminate PFML-PZT-PP. However, in two cases the damage index forms a significant peak only for PFML-PVDF-EP and window size 256, while the use of window size 512 to 2048 results in four out of five specimens with significant peak formation at the moment of complete failure. Finally, there is a configuration where the damage index of laminates based on PZT leads to a significant peak formation in all cases, whereas in the best case four out of five specimens of laminate PFML-PVDF-EP show this peak formation.

##### Characteristic Trend Formation Analysis Results

The trend analysis of the laminates used are shown in Table 6 for the dynamic tests. In case of the standard deviation of the SED, it is evident that PFML-PVDF-EP and PFML-PZT-EP show no trend formation when decibel scaling is used. Also with the laminate PFML-PZT-PP only one specimen in the range of window size 1024 to 8192 forms a characteristic trend, at window size 16,384 it is two specimens and at 32,768 three specimens. Likewise the linear scale usage, one specimen only forms a characteristic trend for laminate PFML-PVDF-EP at window size 256 to 1024 and two specimen from 2048 to 32,768. Similarly, for laminate PFML-PZT-EP only one specimen forms a characteristic trend beginning of windows size 1024, by lower window size usage no specimen forms a trend. In contrast, PFML-PZT-PP shows an increasing number of specimens with trend formation at increasing window size. Furthermore, at the maximum window size all specimens form a characteristic trend while in best case one or two specimen show a trend for laminates PFML-PVDF-EP and PFML-PZT-EP.

The trend analysis of the damage index shows only small differences across all laminates. For laminates PFML-PVDF-EP and PFML-PZT-EP at best two out of five specimens form a characteristic trend. Whereas PFML-PZT-EP shows a trend formation across all window sizes for two specimens, PFML-PVDF-EP only reaches these results in the range of 512 to 2048. PFML-PZT-PP, on the other hand, achieves at best three out of five specimens at window sizes 256 to 2048, beginning at window size 4096 the specimen quantity is reduced to two out of five. Across all laminates, the best results are achieved at window sizes 512 to 2048.

##### Complete Failure Type Analysis Results

Considering the complete failure type in terms of complete failure detection, no significant difference can be recognized, as there is at least one method and configuration for each laminate where all specimens form a significant peak. Equally, the complete failure type in terms of trend analysis shows no difference for the laminates on PZT basis since all specimen of PFML-PZT-PP delaminated and all specimen of PFML-PZT-EP resulted in fibre breakages. In contrast, PFML-PVDF-EP results in three delaminations and two fibre breakages.

By analysing the standard deviation of the SED with linear scaling, one specimen of the delaminations of laminate PFML-PVDF-EP forms a characteristic trend independent of the window size used. Equally, one specimen of the fibre breakages only forms a characteristic trend with similar evaluation parameters at window sizes greater than 2048. Across all specimens of laminate PFML-PVDF-EP no characteristic trend was formed in the trend analysis using the standard deviation of the SED with decibel scale. The analysis results for the damage index are comparable, only one specimen of the delaminations and one specimen of the fibre breakages form a characteristic trend. One difference is represented by the applicable window sizes, in the case of delamination only the window sizes 512 to 2048 lead to a characteristic trend formation, while in the case of a fibre breakage the window sizes 256 to 16,384 show the trend formation.

## 4. Discussion

### 4.1. Detection of Complete Failure

The results show that both the standard deviation of the SED and the damage index *I* represent sufficient methods to detect the time of complete failure. For each laminate at least one parameter set for both presented methods could be identified to detect the time of complete failure for all specimen in both static and dynamic tests scenarios. In the context of using the standard deviation of the SED, the results show that the linear scaling as well as the decibel scaling lead to detection improvements but also to decreases, depending on the specific laminate used. Therefore the scaling type must be considered for each laminate. It is also shown that it is necessary to determine the correct window size for each laminate as, depending on the test scenarios and the methods used, the best results could not be determined for a specific window size. Even though the window sizes from 512 to 2048 give sufficient results across all laminates and methods, corresponding to an observation period of 10 ms to 41 ms. Thus, in both cases the time can be determined by applying the threshold technique, only the determination of the healthy state and the corresponding reference value must be determined for each individual laminate. In summery, the complete failure detection in static test scenarios is going hand in hand with the results of the literature using carbon fibre-reinforced plastic [39,42,43,44], while the dynamic results going along with the results from Saeedifar et.al [45]. Consequently, the hypotheses H1 and H4 are confirmed, as it has been sufficiently demonstrated that both the damage index *I* and the standard deviation of the SED are applicable to determine the time of complete failure.

### 4.2. Characteristic Trend Formation

#### 4.2.1. Static Test Scenario

With regard to the characteristic trend formation, as observed in the case study, the results have to be differentiated. The formation of a characteristic trend in the static tests is observed for all specimens of laminates PFML-PVDF-EP and PFML-PZT-PP, but there was only one specimen for laminate PFML-PZT-EP which formed a characteristic trend by applying damage index methodology. With respect to the standard deviation of the SED only in case of laminate PFML-PVDF-EP all specimens form a characteristic trend, while PFML-PZT-PP forms a characteristic trend in two of three cases and PFML-PZT-EP only in one case. In the context of the damage index use, the results go along with the results from the literature [32,33,34,39]. Furthermore, the significance of the correct window size determination is particularly evident in the characteristic trend formation, as the trend formation is only possible with laminate specific window sizes.

Consequently, Hypothesis H5 can be confirmed partially for laminates PFML-PVDF-EP and PFML-PZT-PP, since for both laminates one method results in a characteristic trend formation for all specimens. Regarding Hypothesis H2, only laminate PFML-PVDF-EP results in a characteristic trend formation for all specimens. Therefore H2 can be confirmed for this laminate only in connection with static test scenarios.

Laminates PFML-PVDF-EP and PFML-PZT-EP are produced according to the same methodology, only the sensors used are different, but significant differences in characteristic trend formation are observed in the static test scenario. The difference in characteristic trend formation of laminates PFML-PVDF-EP and PFML-PZT-EP can be explained by three factors. The first factor, takes into account the type of complete failure, the static test scenario shows that three out of four delaminations form a characteristic trend when using the standard deviation of the SED. Furthermore, all delaminations result in a trend formation when using the damage index. However, no similar behaviour could be found for a glass fibre breakages independent of the methodology. Since all specimens of PFML-PZT-EP result in glass fibre breakages, the type of complete failure represents a possible reason for the observed difference. Secondly, the influence of the sensor type may explain the differences, since PZT and PVDF use the piezoelectric effect but are based on different chemical components leading to sensor specific outputs. The third factor is the amount of specimens, since just three specimens of each laminate have been tested, the effect can be explained by random distribution as well. In conclusion, both methods have the potential to reflect the characteristic trend in a static scenario as long as the parameters are determined and the possible expected type of complete failure is evaluated for the individual laminate.

With regard to Hypothesis H3, the standard deviation of the SED gives the best results for delamination in the static test scenario. Thus, H3 can be partially confirmed for the static tests, but not conclusively clarified, since no direct inheritance comparison of PFML-PZT-EP and PFML-PZT-PP is possible due to a lack of specimens resulting in delamination in PFML-PZT-EP.

#### 4.2.2. Dynamic Test Scenario

In contrast, the results of the dynamic tests rarely show characteristic trend formations for all laminates. The highest amount of trend formation was achieved for laminate PFML-PZT-PP, where all specimens form a trend using the standard deviation of the SED, while laminate PFML-PZT-EP results in one of five and PFML-PVDF-EP in two of five specimens using the same methodology. With regards to the damage index use, laminates PFML-PZT-EP and PFML-PVDF-EP only form a characteristic trend in two of five cases, while laminate PFML-PZT-PP shows in three out of five cases. In consequence, hypotheses H2 and H5 are rejected in the context of the dynamic test scenario. In addition, the delamination results of the static test scenario could not be confirmed in the dynamic test scenarios. Furthermore, no characteristic trend formation for glass fibre breakage could be identified. With regard to Hypothesis H3, no indication of the damage type could be observed. So this hypothesis cannot be confirmed for the dynamic test scenario either.

Nevertheless, in about two to three cases a trend could be observed for all laminates when applying the damage index. This is comparable to the results of the standard deviation of the SED, except for laminate PFML-PZT-PP, where the results of the damage index are behind the standard deviation of the SED. A clarification of the different results of using the damage index in the static and dynamic tests cannot be concluded from the observations made. Therefore, further analyses were conducted examining the observed differences in the damage index.

### 4.3. Damage Index Analysis

The static test results show a general comparability with the results of the damage index by Song, but this is not evident in the dynamic tests. In most cases in literature, an active measurement method is used [33,34,36], so does Song [32]. This allows for a restriction of the frequency ranges to be considered and defines an exact measurement point in time. This represents a difference to the presented dynamic test scenario, since in the present study measurements are made continuously and passively. The piezoelectric effect is generated by continuously increasing and decreasing the pressure on the laminate, leading to a constant variation of the energy generated by the sensors. Furthermore, in the literature mostly large heavy objects [32,33,37,41,46] have been investigated, while this study uses small lightweight components. Consequently, the damage index has to be interpreted differently. The main problem is that the state of health is linked to the dynamics of the universal test machine, thus the damage index oscillates by default. Furthermore, the moment of delamination as well as the glass fibre breakages take place in short energetic increases. Accordingly, the identification of these peaks is difficult as they can be overlaid by the general oscillation. In addition, further disturbances are generated by the noise of the testing machine, which is transmitted as additional vibrations to the laminate and the sensors. An example of a resulting unfiltered signal is shown in Figure 11a,b. For this reason it is necessary to filter the signal to extract the trend.

Furthermore, an association of peak with a glass fibre breakage or delamination has to be ensured. For this reason, the measurements from study 3 are used as references, since here the audio signals were recorded with the cracks of the glass fibre breakages and delaminations. The recorded audio signals are labelled in such a way that each sample of an audible crack is marked as 1, all other samples as 0. However, since not every crack is audible, the force is used as an additional indicator for the static tests and the ratio of displacement to force for the dynamic tests. This distinction is made since in the static tests the displacement increases constantly and has no direct influence. For this reason, any drop in force corresponds to a change in the stiffness of the specimen caused by glass fibre breakage or delamination as it has been shown in [39,42,44,47]. In contrast, the displacement and force changes cyclically in the dynamic tests, so the ratio of both should be constant over time. Any deviation from this indicates a change in the condition of the specimen.

**Figure 10:**
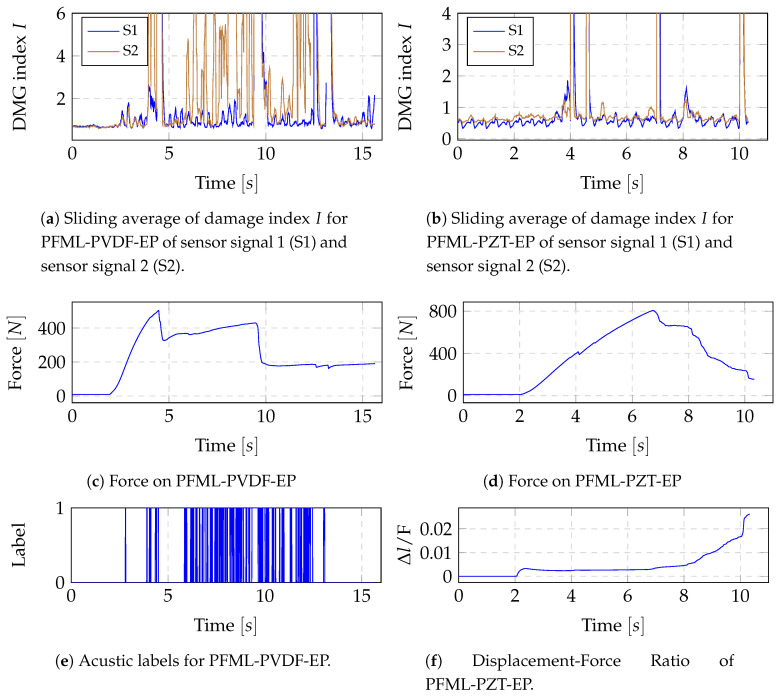
Results of DMG index analysis for one example of PFML-PZT-EP and PFML-PVDF-EP in the static test scenario.

For this reason, in the following a further analysis is carried out to resolve the inconsistencies. The damage index signal has been calculated with a window size of 512 and filtered with a moving average filter of size 10. The results for one specimen of PFML-PVDF-EP and PFML-PZT-EP are shown in Figure 10 for the static test scenario. In the case of laminate PFML-PVDF-EP, it is evident that each drop in force results in a peak of the average damage index. Furthermore, the acoustic label goes along with the peaks of the damage index averages. The same results could be confirmed for all specimens of study 3 in the static test scenario. For the comparison of the specimens from study 2, all specimens of the static test without acoustic label were analysed only regarding the drop in force. The results for one specimen of laminate PFML-PZT-EP are shown in Figure 10b,d,f showing a significant peak of the filtered damage index signal for every drop in force. The described results could be confirmed in the same form for all static test specimens of study 2, including the specimen of laminate PFML-PZT-PP.

**Figure 11:**
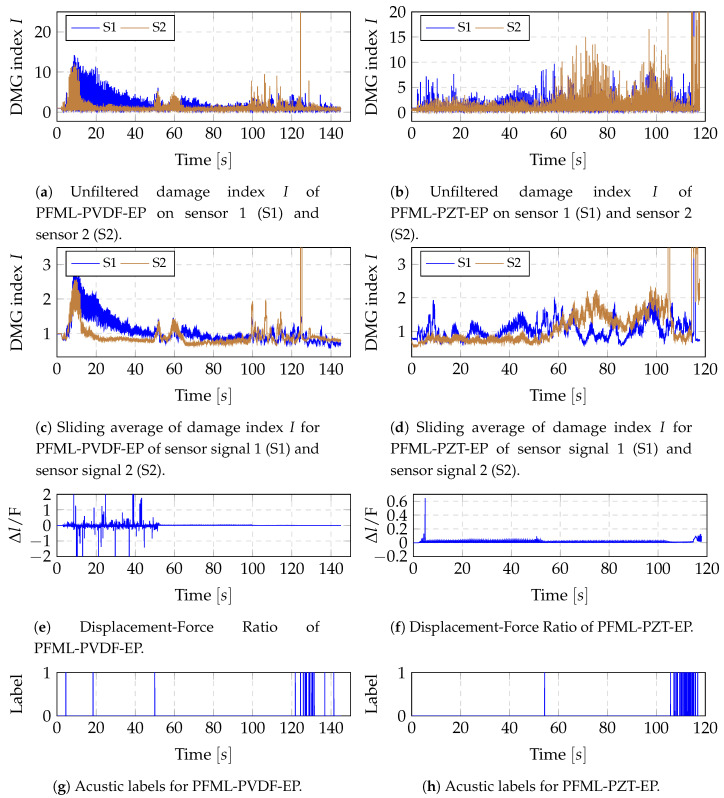
Results of DMG index analysis for one example of PFML-PZT-EP and PFML-PVDF-EP in the dynamic test scenario.

The same procedure was repeated for all specimens of the dynamic test scenario with a window size of 1024 and a moving average of length 15. This distinction was justified by the longer observation time in the dynamic test compared to the static test scenario. The results for one specimen of laminate PFML-PVDF-EP and PFML-PZT-EP are shown in Figure 11. By comparing the filtered damage index signal (Figure 11c,d) with the acoustic labels (Figure 11g,h) it is evident that when a crack is recorded acoustically, an increase in the filtered damage index is associated. Furthermore, when comparing the ratio of displacement and force (Figure 11e,f) with the filtered damage index signal (Figure 11c,d) it is evident, that the range of ratio changes is accompanied by an increase in the filtered damage index, indicating additional class fibre cracks and delaminations. This behaviour could be confirmed for all specimens in study 3.

However, these examples also show that the acoustic labels and the ratio of displacement and force alone are not sufficient to reproduce the process of destruction. An example are the peaks at sensor S2 at time 60 and 100 in Figure 11c, which cannot be explained by the ratio of displacement and force or the acoustic labels. In contrast to the static tests, no conclusion can therefore be drawn about the probes from Study 2.

Another observation is that the signal always returns to the healthy state after a damage occurs leading to peak formations and contradicting to the results from the literature [32,33,34]. One possible explanation of the behaviour can be based on the simple geometry of the specimens and the deformation of the specimens during the experiment. Due to the damage, the stiffness of the specimens decrease, leading to an increased displacement of the laminate. In consequence, the output voltage of the piezoelectric sensors increases, leading to an increased energy level and thus to peak formation. However, over time, the force on the laminate leads to a deformation of the laminate. This deformation causes a reduction in bending and thus a reduction in displacement accompanied by a decreased output voltage of the piezoelectric sensors. As a result, the energy level is reduced, leading to the peak completion and generating a new health state. However, the stiffness is reduced, which should lead to a reduction of the output voltage, accompanied by an energy reduction, but the observations show a comparable energy level as in the healthy state. This in turn can be explained by the piezoelectric effect. Due to the reduced stiffness, the displacement is increased, leading to an increase in the output voltage at the piezoelectric sensors and thus to an increase in energy. In consequence, the increased displacement compensates for the reduced stiffness.

In conclusion, the damage index provides the possibility to detect fibre breakages and delamination in static and dynamic scenarios. A distinction between the two types of damage cannot be conclusively clarified at present. In the static scenario, a characteristic trend formation was observed for all cases of delamination using the damage index, but this could not be confirmed in the dynamic scenarios. Due to the lack of delamination of laminate PFML-PZT-EP, no generalisation can be made with respect to delamination. Due to this reason, Hypothesis H5 can be partially confirmed. Thanks to the detectability of the occurred damages, which would lead to a complete failure in the future, an early warning system can be implemented. However, a direct characteristic trend formation cannot be identified at the current state of the data gathered.

## 5. Conclusions and Future Work

In a case study it was shown that the developed sensory hybrid laminate can be used for condition monitoring. Based on this, a second study with more specimens and variations of the sensor layer and the matrix material was designed. To back up the recorded signals, the second study was repeated in a third study with the recording of an audio signal. For the second and third study, three different aluminium-based PFML types were prepared. A novel piezo-active thermoplastic film based on polypropylene, lead zirconate titanate and carbon nanotubes was used as sensor layer for two PFML types. The matrix material of the glass fibre-reinforced plastic layer was varied between thermoplastic polypropylene and thermoset epoxy resin. The third PFML type uses polyvinylidene fluoride in the sensor layer and epoxy resin in the reinforcement layer. The specimens were produced under laboratory conditions and then tested statically and dynamically in a universal testing machine using a three-point bending test.

The static mechanical properties show that a hybrid laminate with a thermoset matrix achieves the greatest reinforcing effect, but also has the lowest ductility. The failure of the thermoset hybrid laminate is mainly due to fibre breakage. In contrast, the thermoplastic hybrid laminate is more ductile in its failure and maintains a certain level of force over a greater bending distance. The thermoplastic hybrid laminate fails mainly due to delamination.

The evaluation of the dynamic tests shows that the thermoplastic hybrid laminates fail due to delamination even at a low force and after a few cycles. This is attributed to the poor interfacial adhesion between polypropylene and aluminium. The thermoset hybrid laminates, on the other hand, achieve higher forces and withstand more cycles until they fail due to fibre tearing. In both investigations, static and dynamic, no influence of the sensor type on the mechanical behaviour could be determined.

For the analysis of the applicability of the laminates for the SHM sector, two methods were analysed: First, the standard deviation of the spectral energy density (SED) was analysed, motivated by a preliminary study. Furthermore, the damage index as quantification of the energy changes based on wavelet transform was evaluated. The results show that both methods are suitable for detecting the time of complete failure of the laminates in both static and dynamic tests. Furthermore, the results show the necessity to determine an appropriate measurement window size for each individual laminate to ensure a correct failure detection. As an additional criterion, the scaling of the SED was examined, which shows the improvement or deterioration of the results depending on the laminate and measurement window size used.

In addition, both methods were analysed for their suitability for predicting the time of complete failure by analysing the sensor signals for the formation of a characteristic trend. The results show that the damage index for the laminates PFML-PVDF-EP and PFML-PZT-PP forms a characteristic trend in the static test scenario. The same could be observed for the standard deviation of the SED method, only for laminate PFML-PVDF-EP in the static scenario. Furthermore, taking into account the type of damage, indicators were found showing the standard deviation of the SED has a characteristic trend formation in case of delaminations in static scenarios. This behaviour is not observable for the dynamic test scenarios. However, in a further analysis of the damage index, the possibility of detecting delaminations and glass fibre breakages is identified. These are represented by peaks, which are verified by temporal agreement with audible cracks from study 3 and by the correlation of force and displacement. Thus, the damage index could be confirmed as a method to identify damages in the laminates that would lead to a complete failure of the laminate in the future.

In future work, open questions should be clarified. One of these questions is the differentiation of damage types, which was not possible in this studies due to the lack of delaminations for laminate PFML-PZT-EP and the lack of glass fibre breakages for laminate PFML-PZT-PP. For this purpose, it is necessary to identify possibilities to force specific types of damage in order to establish comparability between laminates. Furthermore, the described methods have to be verified for standard laminates such as GLARE, CAPAAL or CAPET [48] in order to address industrial application fields. In addition, the destruction process in the described experiment is always accompanied by a deformation, the influence of which has to be analysed in subsequent studies. These relationships are to be clarified in future work by means of numerical simulation. Furthermore, it is planned to determine the parameters for damage models more precisely by means of the presented SHM system. One possible methodology would be represented by further detailed wavelet transform analysis to identify a specific frequency band of interest in order to determine a minimum sampling rate for the implementation of mobile embedded systems. Furthermore, it has to be investigated whether the specific frequency bands representing failure types as it has been shown in [39,43]. The presented analysis showed that the combination of audible cracks and the ratio of displacement and force are good indicators for damages, but do not explain the whole signal. For their further analysis, methods have to be developed to detect partial delaminations and smaller cracks in real time and verify them with the curve of the sensor signals. One possible method for verification could be an endurance test in which the specimen is examined at regular intervals by computer tomography.

## Figures and Tables

**Table 1:** Summary of the results from the static three-point bending tests for the thermoplastic composites.

Composite	Max. Force [N]	Bending Distance [mm]	Strengthening *S*	Ductility *D*
Al-PZT	174.48	13.97	100%	100%
PFML-PZT-PP	452.12	12.00	259%	86%
PFML-PZT-EP	752.76	4.02	431.40%	28.80%
PFML-PVDF-EP	779.7	3.78	446.90%	27.00%

**Table 2:** Analysis results for complete failure detection by the standard deviation of the SED (STD) and the damage index (DMG) as a function of scaling and measurement window size for the amount of specimens (numbers in brakes show the retaliative quantity of specimens).

		Window Size in Samples							
**Laminate**	**Method**	**256**	**512**	**1024**	**2048**	**4096**	**8192**	**16,384**	**32,768**
**PFML-** **PVDF-EP**	STD-linear	3 (100%)	3 (100%)	3 (100%)	3 (100%)	3 (100%)	2 (66.6%)	-	-
	STD-dB	3 (100%)	3 (100%)	3 (100%)	3 (100%)	3 (100%)	3 (100%)	-	-
	DMG	3 (100%)	3 (100%)	3 (100%)	3 (100%)	3 (100%)	3 (100%)	3 (100%)	2 (66.6%)
**PFML-** **PZT-EP**	STD-linear	2 (66.6%)	2 (66.6%)	2 (66.6%)	2 (66.6%)	2 (66.6%)	-	-	-
	STD-dB	2 (66.6%)	2 (66.6%)	2 (66.6%)	3 (100%)	3 (100%)	-	-	-
	DMG	3 (100%)	3 (100%)	3 (100%)	3 (100%)	2 (66.6%)	2 (66.6%)	2 (66.6%)	2 (66.6%)
**PFML-** **PZT-PP**	STD-linear	3 (100%)	3 (100%)	3 (100%)	3 (100%)	3 (100%)	2 (66.6%)	2 (66.6%)	-
	STD-dB	-	-	3 (100%)	3 (100%)	3 (100%)	2 (66.6%)	2 (66.6%)	-
	DMG	3 (100%)	3 (100%)	3 (100%)	3 (100%)	2 (66.6%)	2 (66.6%)	-	-

**Table 3:** Analysis results for trend formation by the standard deviation of the SED (STD) and the damage index (DMG) as a function of scaling and measurement window size for the amount of specimens (numbers in brakes show the retaliative quantity of specimens).

		Window Size in Samples							
**Laminate**	**Method**	**256**	**512**	**1024**	**2048**	**4096**	**8192**	**16,384**	**32,768**
**PFML-** **PVDF-EP**	STD-linear	-	-	-	1 (33.3%)	2 (66.6%)	2 (66.6%)	-	-
	STD-dB	-	-	-	1 (33.3%)	2 (66.6%)	3 (100%)	-	-
	DMG	2 (66.6%)	1 (33.3%)	3 (100%)	3 (100%)	2 (66.6%)	3 (100%)	3 (100%)	1 (33.3%)
**PFML-** **PZT-EP**	STD-linear	-	-	-	-	1 (33.3%)	-	-	-
	STD-dB	-	-	-	-	1 (33.3%)	1 (33.3%)	-	-
	DMG	1 (33.3%)	1 (33.3%)	1 (33.3%)	1 (33.3%)	1 (33.3%)	1 (33.3%)	-	-
**PFML-** **PZT-PP**	STD-linear	-	-	-	-	-	-	-	-
	STD-dB	-	-	1 (33.3%)	1 (33.3%)	2 (66.6%)	2 (66.6%)	2 (66.6%)	-
	DMG	2 (66.6%)	3 (100%)	2 (66.6%)	-	-	1 (33.3%)	-	-

**Table 4:** Summary of the results from the dynamic three-point bending tests for the thermoplastic composites.

Composites	Max. Force [N]	Max. Cycle
PFML-PZT-PP	396.26	234
PFML-PZT-EP	774.51	472
PFML-PVDF-EP	730.67	444

**Table 5:** Analysis results for complete failure detection by the standard deviation of the SED (STD) and the damage index (DMG) as a function of scaling and measuring window size for the amount of specimens (numbers in brakes show the retaliative quantity of specimens).

		Window Size in Samples							
**Laminate**	**Method**	**256**	**512**	**1024**	**2048**	**4096**	**8192**	**16,384**	**32,768**
**PFML-** **PVDF-EP**	STD-linear	5 (100%)	5 (100%)	5 (100%)	5 (100%)	5 (100%)	5 (100%)	5 (100%)	4 (80%)
	STD-dB	5 (100%)	5 (100%)	5 (100%)	5 (100%)	5 (100%)	5 (100%)	5 (100%)	4 (80%)
	DMG	2 (40%)	4 (80%)	4 (80%)	4 (80%)	3 (60%)	3 (60%)	3 (60%)	3 (60%)
**PFML-** **PZT-EP**	STD-linear	5 (100%)	5 (100%)	5 (100%)	5 (100%)	5 (100%)	5 (100%)	5 (100%)	5 (100%)
	STD-dB	4 (80%)	5 (100%)	5 (100%)	5 (100%)	5 (100%)	5 (100%)	5 (100%)	5 (100%)
	DMG	5 (100%)	5 (100%)	5 (100%)	5 (100%)	5 (100%)	5 (100%)	5 (100%)	5 (100%)
**PFML-** **PZT-PP**	STD-linear	4 (80%)	4 (80%)	4 (80%)	4 (80%)	4 (80%)	4 (80%)	4 (80%)	4 (80%)
	STD-dB	1 (20%)	3 (60%)	3 (60%)	2 (40%)	2 (40%)	3 (60%)	3 (60%)	3 (60%)
	DMG	5 (100%)	5 (100%)	5 (100%)	5 (100%)	4 (80%)	4 (80%)	4 (80%)	4 (80%)

**Table 6:** Analysis results for trend formation detection by the standard deviation of the SED (STD) and the damage index (DMG) as a function of scaling and measuring window size or the amount of specimens (numbers in brakes show the retaliative quantity of specimens).

		Window Size in Samples							
**Laminate**	**Method**	**256**	**512**	**1024**	**2048**	**4096**	**8192**	**16,384**	**32,768**
**PFML-** **PVDF-EP**	STD-linear	1 (20%)	1 (20%)	1 (20%)	2 (40%)	2 (40%)	2 (40%)	2 (40%)	2 (40%)
	STD-dB	-	-	-	-	-	-	-	-
	DMG	1 (20%)	2 (40%)	2 (40%)	2 (40%)	1 (20%)	1 (20%)	1 (20%)	-
**PFML-** **PZT-EP**	STD-linear	-	-	1 (20%)	1 (20%)	1 (20%)	1 (20%)	1 (20%)	1 (20%)
	STD-dB	-	-	-	-	-	-	-	-
	DMG	2 (40%)	2 (40%)	2 (40%)	2 (40%)	2 (40%)	2 (40%)	2 (40%)	2 (40%)
**PFML-** **PZT-PP**	STD-linear	2 (40%)	2 (40%)	2 (40%)	3 (60%)	4 (80%)	4 (80%)	4 (80%)	5 (100%)
	STD-dB	-	-	1 (20%)	1 (20%)	1 (20%)	1 (20%)	2 (40%)	3 (60%)
	DMG	3 (60%)	3 (60%)	3 (60%)	3 (60%)	2 (40%)	2 (40%)	2 (40%)	2 (40%)

## Abbreviations

The following abbreviations are used in this manuscript.

AL-PZT	Aluminium sheet with PZT/PP film sensor layer
CNT	Carbon Nanotubes
CWT	Continuous Wavelet Transform
EP	Epoxy Resin
FBG	Fibre Bragg Grating
FML	Fibre Metal Laminates
GFRP	Glass Fibre-Reinforced Plastic
MMD	Multi-material Design
NDT	Non-destructive Testing
PFML	Piezo-active Fibre Metal Laminate
PFML-PVDF-EP	aluminium-based PFML with PVDF foil sensor layer and glass fibre epoxy reinforcement
PFML-PZT-EP	aluminium-based PFML with PZT/PP foil sensor layer and glass fibre epoxy reinforcement
PFML-PZT-PP	aluminium-based PFML with PZT/PP foil sensor layer and glass fibre polypropylene reinforcement
PP	Polypropylene
PVDF	Polyvinlyliden Fluoride
PZT	Lead Zirconate Titanate
SED	Spectral Energy Density
SHM	Structural Health Monitoring

## References

[1] Heimbs S., Bergmann T., Schueler D., Toso-Pentecote N. (2014). High velocity impact on preloaded composite plates. Compos. Struct..

[2] García-Martín J., Gómez-Gil J., Vázquez-Sánchez E. (2011). Non-destructive techniques based on eddy current testing. Sensors.

[3] Cramer K. (2018). Current and future needs and research for composite materials NDE. Proceedings Volume 10596, Behavior and Mechanics of Multifunctional Materials and Composites XII.

[4] Francis D., Tatam R., Groves R. (2010). Shearography technology and applications: A review. Meas. Sci. Technol..

[5] Gholizadeh S. (2016). A review of non-destructive testing methods of composite materials. Procedia Struct. Integr..

[6] Giurgiutiu V. (2015). 16–Structural health monitoring (SHM) of aerospace composites. Polymer Composites in the Aerospace Industry.

[7] Kappel E., Prussak R., Wiedemann J. (2019). On a simultaneous use of fiber-Bragg-gratings and strain-gages to determine the stress-free temperature Tsf during GLARE manufacturing. Compos. Struct..

[8] Sousa J., Marques J., Garcia M., Infante V., Amaral P. (2020). Mechanical characterization of sandwich composites with embedded sensors. Eng. Fail. Anal..

[9] Kuang K., Cantwell W., Zhang L., Bennion I., Maalej M., Quek S. (2005). Damage monitoring in aluminum-foam sandwich structures based on thermoplastic fibre-metal laminates using fibre Bragg gratings. Compos. Sci. Technol..

[10] Du C., Dutta S., Kurup P., Yu T., Wang X. (2020). A review of railway infrastructure monitoring using fiber optic sensors. Sens. Actuators Phys..

[11] Gunes O. (2013). 5–Failure modes in structural applications of fiber-reinforced polymer (FRP) composites and their prevention. Developments in Fiber-Reinforced Polymer (FRP) Composites for Civil Engineering.

[12] Tuloup C., Harizi W., Aboura Z., Meyer Y., Khellil K., Lachat R. (2019). On the use of in-situ piezoelectric sensors for the manufacturing and structural health monitoring of polymer-matrix composites: A literature review. Compos. Struct..

[13] Jung K.C., Chang S.H. (2019). Performance evaluation of smart grid fabrics comprising carbon dry fabrics and PVDF ribbon sensors for structural health monitoring. Compos. Part B Eng..

[14] Memmolo V., Monaco E., Boffa N., Maio L., Ricci F. (2018). Guided wave propagation and scattering for structural health monitoring of stiffened composites. Compos. Struct..

[15] Hartwig M., Gaitzsch M., Großmann T., Heinrich M., Kroll L., Gessner T., Baumann R. (2016). Investigation on an inkjet printed passive sensor for wireless ice detection onwind rotor blades. J. Imaging Sci. Technol..

[16] Großmann T., Hartwig M., Heinrich M., Decker R., Symmank C., Schmidt A., Kurth S., Götze U., Baumann R., Kroll L. Realization of Sensitive Functionality by the Integration of Electromagnetic Resonators in Composite Materials. Proceedings of the 3rd International MERGE Technologies Conference (IMTC).

[17] Ullmann F., Decker R., Graf A., Kräusel V., Heinrich M., Hardt W., Kroll L., Landgrebe D. (2017). Continuous Manufacturing of Piezoceramic Hybrid Laminates for Functionalised Formed Structural Components. Technol. Lightweight Struct..

[18] Kräusel V., Graf A., Heinrich M., Decker R., Caspar M., Kroll L., Hardt W., Göschel A. (2015). Development of hybrid assembled composites with sensory function. CIRP Ann. Manuf. Technol..

[19] Nestler D. (2014). Beitrag zum Thema: Verbundwerkstoffe-Werkstoffverbunde. Status quo und Forschungsansätze.

[20] Zopp C., Nestler D., Tröltzsch J., Trautmann M., Nendel S., Wangner G., Nendel W., Kroll L. (2017). CATPUAL-An Innovative and High-Performance Hybrid Laminate with Carbon Fibre-Reinforced Thermoplastic Polyurethane. Key Eng. Mater..

[21] Ulke-Winter L., Klärner M., Kroll L. (2013). Determining the damping behavior of fibre reinforced composites-A new approach to find mathematical relationships of data sets. Compos. Struct..

[22] Kräusel V., Graf A., Decker R., Ullmann F., Heinrich M., Landgrebe D., Kroll L., Hardt W. (2017). Mass production enabled manufacturing and measurment technologies for hybrid laminates with sensory function. Proceedings of the 3rd International MERGE Technologies Conference–IMCT 2017 Lightweight Structures.

[23] Doerffel C., Decker R., Heinrich M., Tröltzsch J., Spieler M., Nendel W., Kroll L. (2017). Polypropylene based piezo ceramic compounds for micro injection molded sensors. Key Eng. Mater..

[24] Decker R., Heinrich M., Reindel P., Sockol S., Päßler E., Kroll L. (2018). Functionalized Compounds for Micro-Injection Molded Piezo Modules (*μ*IMP-Modules) and Their Electrical Contacting. Adv. Eng. Mater..

[25] Kroll L. (2019). Technologiefusion für multifunktionale Leichtbaustrukturen.

[26] Schmidt R., Graf A., Decker R., Kräusel V., Hardt W., Landgrebe D., Kroll L. (2018). Hybrid laminate for haptic input device with integrated signal processing. Appl. Sci..

[27] Graf A., Decker R., Schmidt R., Kräusel V., Kroll L., Hardt W. (2019). Haptic input devices with intelligent Continuous Manufacturing of Piezoceramic Hybrid Laminates for Functionalised Formed Structural Components. Technol. Lightweight Struct..

[28] (2017). ASTM D7774-17 Standard Test Method for Flexural Fatigue Properties of Plastics. https://shop.standards.ie/EMEA/Details.aspx?ProductID=1919391.

[29] Mast P.W., Michopoulos J.G., Badaliance R., Chaskelis H.H. (1994). Dissipated energy as the means for health monitoring of smart structures. Smart Structures and Materials 1994: Smart Sensing, Processing, and Instrumentation. Int. Soc. Opt. Photonics.

[30] Samuel P.D., Pines D.J. (2001). Classifying helicopter gearbox faults using a normalized energy metric. Smart Mater. Struct..

[31] Staszewski W.J., Robertson A.N. (2007). Time–frequency and time–scale analyses for structural health monitoring. Philos. Trans. R. Soc. A Math. Phys. Eng. Sci..

[32] Song G., Gu H., Mo Y., Hsu T., Dhonde H. (2007). Concrete structural health monitoring using embedded piezoceramic transducers. Smart Mater. Struct..

[33] Zeng L., Parvasi S.M., Kong Q., Huo L., Li M., Song G. (2015). Bond slip detection of concrete-encased composite structure using shear wave based active sensing approach. Smart Mater. Struct..

[34] Xu K., Ren C., Deng Q., Jin Q., Chen X. (2018). Real-time monitoring of bond slip between GFRP bar and concrete structure using piezoceramic transducer-enabled active sensing. Sensors.

[35] Kong Q., Robert R.H., Silva P., Mo Y. (2016). Cyclic crack monitoring of a reinforced concrete column under simulated pseudo-dynamic loading using piezoceramic-based smart aggregates. Appl. Sci..

[36] Han F., Jiang J., Xu K., Wang N. (2019). Damage detection of common timber connections using piezoceramic transducers and active sensing. Sensors.

[37] Gómez M.J., Corral E., Castejon C., García-Prada J.C. (2018). Effective crack detection in railway axles using vibration signals and WPT energy. Sensors.

[38] Yu Y., Dackermann U., Li J., Niederleithinger E. (2019). Wavelet packet energy–based damage identification of wood utility poles using support vector machine multi-classifier and evidence theory. Struct. Health Monit..

[39] Saeedifar M., Najafabadi M.A., Zarouchas D., Toudeshky H.H., Jalalvand M. (2018). Barely visible impact damage assessment in laminated composites using acoustic emission. Compos. Part B Eng..

[40] Barile C., Casavola C., Pappalettera G., Vimalathithan P.K. (2019). Damage characterization in composite materials using acoustic emission signal-based and parameter-based data. Compos. Part B Eng..

[41] Qin F., Kong Q., Li M., Mo Y., Song G., Fan F. (2015). Bond slip detection of steel plate and concrete beams using smart aggregates. Smart Mater. Struct..

[42] Barile C., Casavola C., Moramarco V., Pappalettere C., Vimalathithan P.K. (2020). Bonding Characteristics of Single-and Joggled-Lap CFRP Specimens: Mechanical and Acoustic Investigations. Appl. Sci..

[43] Barile C., Casavola C., Pappalettera G., Pappalettere C., Vimalathithan P.K. (2020). Detection of Damage in CFRP by Wavelet Packet Transform and Empirical Mode Decomposition: An Hybrid Approach. Appl. Compos. Mater..

[44] Samborski S., Gliszczynski A., Rzeczkowski J., Wiacek N. (2019). Mode I Interlaminar Fracture of Glass/Epoxy Unidirectional Laminates. Part I: Experimental Studies. Materials.

[45] Saeedifar M., Mansvelder J., Mohammadi R., Zarouchas D. (2019). Using passive and active acoustic methods for impact damage assessment of composite structures. Compos. Struct..

[46] Liao W.I., Chiu C.K. (2019). Seismic health monitoring of a space reinforced concrete frame structure using piezoceramic-based sensors. J. Aerosp. Eng..

[47] Bai R., Guo J., Lei Z., Liu D., Ma Y., Yan C. (2019). Compression after impact behavior of composite foam-core sandwich panels. Compos. Struct..

[48] Wielage B., Nestler D., Steger H., Kroll L., Tröltzsch J., Nendel S., Fathi M., Holland A., Ansari F., Weber C. (2011). CAPAAL and CAPET–New Materials of High-Strength, High-Stiff Hybrid Laminates. Integrated Systems, Design and Technology 2010.

